# Adaptive Distance Metric Learning for Diffusion Tensor Image Segmentation

**DOI:** 10.1371/journal.pone.0092069

**Published:** 2014-03-20

**Authors:** Youyong Kong, Defeng Wang, Lin Shi, Steve C. N. Hui, Winnie C. W. Chu

**Affiliations:** 1 Department of Imaging and Interventional Radiology, The Chinese University of Hong Kong, Shatin, New Territories, Hong Kong, China; 2 Research Center for Medical Image Computing, The Chinese University of Hong Kong, Shatin, New Territories, Hong Kong, China; 3 The Chinese University of Hong Kong Shenzhen Research Institute, Shenzhen, China; 4 Department of Biomedical Engineering and Shun Hing Institute of Advanced Engineering, The Chinese University of Hong Kong, Shatin, New Territories, Hong Kong, China; 5 Shenzhen Institutes of Advanced Technology, Chinese Academy of Sciences, Shenzhen, China; Vanderbilt University, United States of America

## Abstract

High quality segmentation of diffusion tensor images (DTI) is of key interest in biomedical research and clinical application. In previous studies, most efforts have been made to construct predefined metrics for different DTI segmentation tasks. These methods require adequate prior knowledge and tuning parameters. To overcome these disadvantages, we proposed to automatically learn an adaptive distance metric by a graph based semi-supervised learning model for DTI segmentation. An original discriminative distance vector was first formulated by combining both geometry and orientation distances derived from diffusion tensors. The kernel metric over the original distance and labels of all voxels were then simultaneously optimized in a graph based semi-supervised learning approach. Finally, the optimization task was efficiently solved with an iterative gradient descent method to achieve the optimal solution. With our approach, an adaptive distance metric could be available for each specific segmentation task. Experiments on synthetic and real brain DTI datasets were performed to demonstrate the effectiveness and robustness of the proposed distance metric learning approach. The performance of our approach was compared with three classical metrics in the graph based semi-supervised learning framework.

## Introduction

The emerging diffusion tensor imaging (DTI) has been increasingly applied to study the structure and function of the human brain [1], [2]. This noninvasive imaging modality can capture the tissue microstructure by measuring the diffusion information of water molecules [3], [4]. The wealthy information is able to differentiate complex anatomical structures, which are difficult to be distinguished by conventional imaging modalities [5]. Thereby, DTI has recently drew more interest in the segmentation of several tissues [6] and white matter tracts [7], [8]. High quality segmentation is of key importance in biomedical research and clinical application [9], [10]. It is crucial to develop an efficient and effective method for accurate segmentation of interested structures in DTI.

The performance of image segmentation critically depends on the choice of an appropriate distance measure. A number of metrics have been developed to differentiate diffusion tensors, including Euclidean [11], J-divergence [12] and geodesic metrics [13], [14]. They provide an overall distance with unknown or fixed contribution of geometry and orientation distances. However, the two types of distances are not always equally relevant for segmentation of different tissues. For instance, tensors belonging to the corpus callosum and the cingulum, have similar geometry shapes but completely different orientations [15]. Therefore, the traditional metrics, which give fixed weights of geometry and orientation distances, may result in a relatively low discrimination capability.

In recent years, a large amount of efforts have been spent on constructing discriminative metrics by selecting relevant features for different segmentation applications. Fusion of the geometrical distances was able to obtain accurate segmentation of white matter, grey matter and cerebrospinal fluid in the human brain [16], [17]. The orientation distance has been utilized to facilitate automatic identification of different nucleus in the thalamus of human brain [6], [18]. Recently, Luis-Garc’ıa et al. [15] and Gahm et al. [19] proposed to construct a novel distance metric by manually weighting the geometry and orientation distances. The manual tuning of weights obtained accurate results in the white matter structures segmentation from the human brain [15] and the tissues extraction from the human cardiac [19]. Sufficient prior knowledge is required to guide the distance selection for different segmentation tasks. Tuning parameters should be performed on a large number of datasets. Moreover, the parameters gained from datasets in one group may not be optimal for another one due to difference in subject anatomies and imaging parameters. Instead of predefining a distance function, it is more appealing to automatically learn a distance metric for each specific DTI segmentation task.

Distance metric learning has attracted considerable amount of interest in the research of machine learning [20], [21], image processing [22] and pattern recognition [23], [24]. It has been successfully applied to obtain discriminative metrics for several applications, such as image retrieval [25], [26] and classification [27]. The target is to learn an appropriate distance measure from the supervisory data. Thereafter, the examples from the same class are close to each other, and while examples from different classes are set to a large distance [28]. Inspired by this idea, distance metric learning can be extended to differentiate diffusion tensors. To the best of our knowledge, we for the first time propose to utilize distance metric learning to train an adaptive metric between diffusion tensors for DTI segmentation. We hope that more discriminative distances could be achieved to obtain correct and accurate segmentation results.

In this study, we proposed to learn an adaptive distance metric in a graph based semi-supervised learning model for DTI segmentation. Our aim was to learn a nonlinear mapping of an original distance to maintain the label of voxels by their distances from the supervisor information. An original distance vector was first formulated by combining both geometry and orientation distances derived from diffusion tensors. The distance metric and labels of voxels were then optimized in a graph based semi-supervised learning model. Finally, the optimization task was efficiently solved by an iterative gradient descent approach. With our proposed approach, an adaptive metric could be created for each specific segmentation task to achieve a correct and accurate segmentation result. We validated the proposed method on both synthetic and real brain DTI datasets from nine subjects. The performance of our approach was compared with three classical metrics in the graph based semi-supervised learning framework.

## Materials and Methods

### Synthetic Dataset

**Figure 1:**
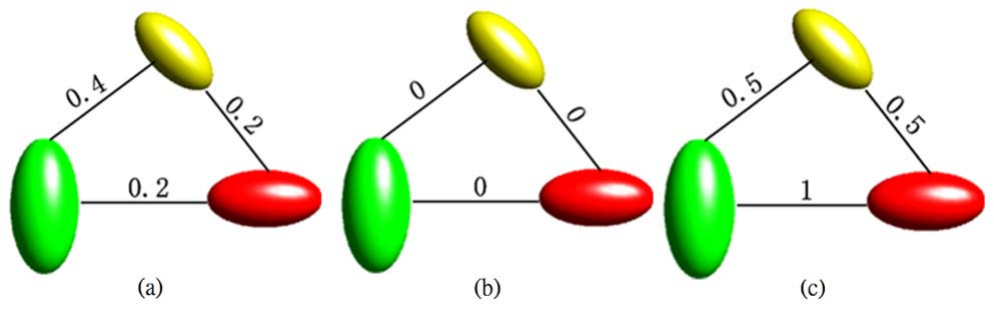
Segmentation results of the synthetic dataset: (a) user input labels, (b) segmentation results of Euclidean, J-divergence, geodesic metrics, (c) segmentation results of our approach. The red color bar represented the label of the interested regions and the other two green bars are the labels of the background.

A specific synthetic dataset was created to validate the proposed segmentation approach. The synthetic tensor field was a 15×15 lattice, and the region of interest was different from the background in the geometry properties. The synthetic diffusion tensors are visualized as ellipses, as shown in figure 1(a). Each ellipse is constructed using eigenvectors of the tensor as axes and the color represents the principal orientation.

Noisy tensor fields were generated by adding noise to the clean synthetic tensor field. We utilized Stejkal-Tanner equation to generate 12 clean diffusion weighted images (DWI) with b values of 1000 s/mm^2^ and one baseline image with b values of 0 s/mm^2^. Rician noise was then simulated on each DWI with signal to noise (SNR) of 10, 15 and 20 [29], [30]. The noisy synthetic tensor fields were finally estimated at the three noise levels.

### Subjects and DTI Acquisition

Written informed consent forms were obtained from nine healthy subjects recruited from a local tertiary teaching hospital. Ethical approval was obtained from the Ethics Committee in the Chinese University of Hong Kong. All subjects underwent a MRI scanning in a 3 Tesla scanner with an eight-channel Sense head coil (Achieva, Philips Medical Systems) at the Prince of Wales Hospital at Hong Kong. For each subject, brain DTI was acquired with a single-shot spin-echo echo-planar pulse sequence with the following parameters, repetition time = 8667 ms, echo time = 60 ms, field of view = 224×224 mm^2^, flip angle = 90^o^, NEX = 1, matrix = 112×109, slice = 70, slice thickness = 2 mm, gap = 2 mm. After reconstruction, 70 axial images were zero-padded and interpolated to 224×224 with voxel size of 1×1×2 mm^3^. In DTI data acquisition, images at b values of 1000s/mm^2^ were acquired along 32 directions of diffusion gradients, and one image at b value of 0s/mm^2^ was acquired as the baseline image.

### Original Discriminative Distance Vector

The aim of distance metric learning is to learn a linear or nonlinear mapping of an original distance to produce an appropriate metric. An original discriminative distance is hence crucial to characterize the difference between voxels. Different from traditional scalar medical images, each voxel is assigned to a 3×3 symmetric positive definite diffusion tensor [31], [32]. The diffusion tensor can be decomposed into a system of three eigenvalues and corresponding eigenvectors, which represent tensor geometry and orientation respectively. The original distance vector is formulated by combining both geometry and orientation distances.

Based on the three eigenvalues, several geometrical characteristics have been developed to capture the water diffusivity and anisotropy properties of the diffusion tensor [33]. The most commonly utilized properties are mean diffusivity (MD), fractional anisotropy (FA) and volume ratio (VR) [10], [34]. The definitions of these features are given as follows
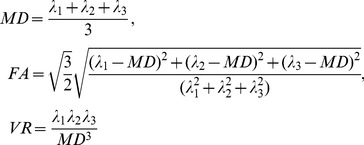
(1)where 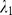, 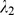 and 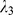 are the three eigenvalues. MD measures the overall diffusivity, and FA and VR characterize the anisotropy. They have the ability to distinguish different tissues, which have been frequently demonstrated in DTI analysis and segmentation tasks [16], [17], [35], [36]. They are grouped together to form the geometrical feature vector 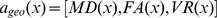. Elements of the feature vector are then normalized to [0 1] prior to distance calculation. The geometrical distance vector is hence formulated as
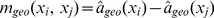
(2)where 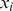 and 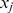 denote two voxels, 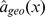 is the normalized geometrical feature vector.

The eigenvector 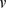 corresponding to the largest eigenvalue of diffusion tensor is characterized as the principal orientation. The field of this vector can indicate the homogeneity of fiber orientations in the white matter regions. This important feature has been demonstrated to be able to differentiate several anatomical structures [6], [18] and white matter tracts [37]. Different from the scalar geometry distances, the orientation distance is computed by the rotation angle between principal eigenvectors. Since an eigenvector has an arbitrary sign, 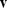 and 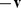 correspond to the same orientation but the opposite direction. The orientation distance is thus defined as the minimum rotation between the principal eigenvectors. The orientation distance is normalized to [0 1] as
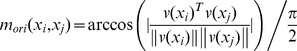
(3)


The geometry distance vector and orientation distance are thus combined together to formulate the original distance vector 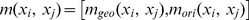.

**Figure 2:**
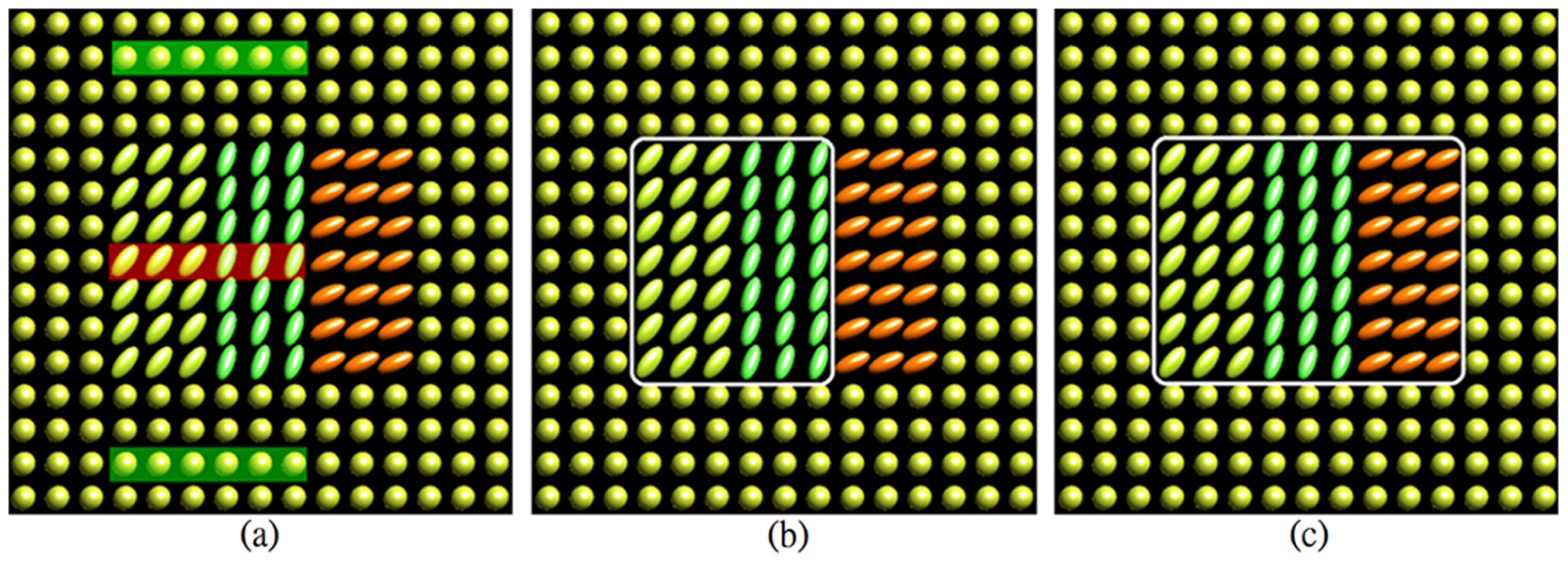
Euclidean distances of different properties between diffusion tensors, (a) mean diffusion (MD), (b) fractional anisotropy (FA) and (c) principal orientation.

Figure 2 illustrates Euclidean distances of MD, FA and principal orientation between diffusion tensors. Each tensor is visualized as an ellipse with eigenvectors of the corresponding diffusion tensor as axes. The color denotes the principal orientation of the diffusion tensor. The tensors have the same FA value with different MD values and principal orientations. Selecting different features to characterize the distance leads to different classifications. Therefore, it is essential to choose a proper distance metric to differentiate diffusion tensors for each segmentation task.

### Distance Metric Learning

Selecting an appropriate metric between voxels is fundamental to the DTI segmentation tasks. Segmentation methods by predefined metrics require sufficient prior knowledge and tuning parameters on a large number of datasets. To overcome these disadvantages, distance metric learning will be introduced to automatically learn an optimal metric for each segmentation application.

Existing distance metric learning approaches can be classified into categories of unsupervised, supervised and semi-supervised learning [28]. Unsupervised learning methods learn the metric without labels. Unfortunately, under unsupervised learning models, the distance metric learning tasks become an ill-conditioned problem with no well-defined optimization criteria [38], [39]. For example, one widely utilized unsupervised learning method, named principal component analysis, simply reweighs the features and may end up turning a relevant feature into an irrelevant one [40].

In supervised and semi-supervised learning models, labeled training data is available to provide supervisory information, and typically limited number of labeled data with large quantity of unlabeled data is considered as semi-supervised learning [20], [23]. Class labels from users provide pairwise constraints to learn the metric. An optimal metric is learned with keeping the examples from same class close to each other, while separating the data elements from different class [25], [41]. Compared to the supervised learning, semi-supervised learning has the capability to avoid the over fitting problem when the labeled training data is insufficient [21]. For the DTI segmentation application, a limited number of voxels can be given as the labels of the foreground and background from users. Therefore, a graph based semi-supervised learning approach will be adopted to learn adaptive distance metrics for DTI segmentation.

An undirected weighted graph 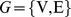 is first constructed for the DTI image. The vertices V of the graph are voxels of the images, and E represents the edges between these vertices. If two voxels 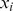 and 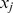 are spatial neighbors, an edge 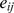 exists to connect them in the graph. The weight 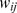 of each edge is the similarity between tensors at voxel 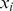 and 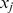. In the traditional graph based approaches for DTI segmentation [19], [42], [43], the weight is defined by a Gaussian kernel function of the predefined distance as
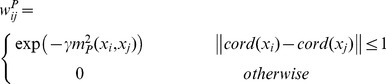
(4)where 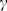 is the parameter of Gaussian function, 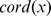 is the coordinate of voxel 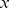, 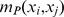 is the predefined distance metric between voxel 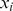 and voxel 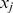. Two voxels are connected with spatial distance between their coordinates less than or equal to 1. The commonly utilized predefined metric are the Euclidean, J-divergence or geodesic metrics [42], [43]. They are defined as



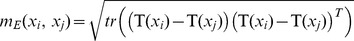
(5)

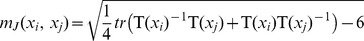
(6)




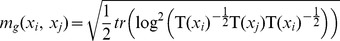
(7)where 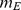, 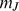 and 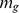 are definitions of the Euclidean, J-divergence or geodesic metrics. 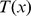 is the diffusion tensor at location x and 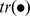 is the trace of matrix. Besides, the predefined distance can also be a linear mapping of the original distance. However, the weights of different type of distances are manually tuned in the work of [19]. In this paper, we for the first time focus on the basic Mahalanobis distance metric to learn a kernel metric to define the weight of edge as
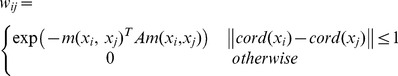
(8)where 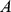 is a positive semi-definite matrix. Let 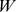 be the graph weight matrix whose element is 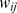 and 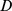 be a diagonal matrix whose 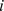-th diagonal element is 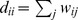.

Labeled voxels of interested anatomical structures and background in DTI images are provided as the supervisory information. The labels are modeled as 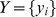, where 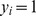 if voxel at 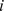 is marked as the interested anatomical structure, and −1 if marked as the background surrounding structures. In traditional graph based semi-supervised learning approaches, Gaussian kernel function of the predefined metric as equation (4) was utilized to compute the edges of graph. From the idea of learning local and global consistency by Zhou et al. [44], labels of voxels represented by 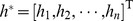
can be learned by minimizing the following cost function
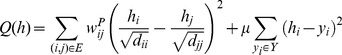
(9)where 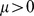 is the regularization parameter. The first term of the right-hand side denotes the smoothness restraint, which signifies that there should be not too much change between nearby voxels. The second term represents the fitting constraints, which implies that the result of a good segmentation should not be changed too much from the user initial label assignment. A positive parameter 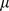 is introduced to control trade-off between these two terms. For the image segmentation tasks, the labels provided by users are generally assumed to be correct. The second term can be treated as hard constraints. The optimization of voxels labeling can be transferred to a minimization problem as



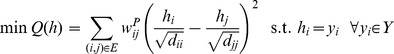
(10)The comparison experiments utilized three classical metrics, including Euclidean, J-divergence and geodesic distances, in this graph based semi-supervised learning method.

The aim of distance metric learning is to achieve an optimal kernel metric 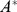 to maintain the label information of voxels by their distances. With such metric, the voxels in the same label are close to each other, whereas the voxels in the different labels have large distances. To integrate metric learning and label training, the predefined distance is replaced by the above weight computed from the Mahalanobis distance metric as equation (8). The distance metric learning and label learning can be simultaneously obtained as
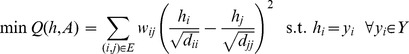
(11)with 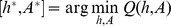.

### Numerical Solution

It is difficult to optimize 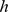 and 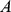 at the same time. Therefore, the optimization is solved using an iterated alternative approach. We first fix the metric 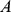 to update the class label 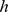, and then update the metric 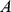 by fixing the class label 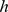.

At the class label update stage, the optimal solution 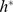 can be found with a fixed metric 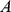. The minimization of equation (11) can be written in another way as
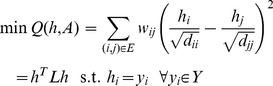
(12)where 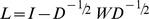 is the normalized Laplacian matrix [45] of the constructed graph 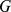. The minimization problem is then turned to a quadratic programming problem. It can be efficiently solved with the interior point method to achieve the global optimal solution 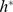 [46].

At the metric update stage, it is difficult to facilitate a closed form solution to the optimization task. The gradient descent approach is thus adopted to solve this problem. The derivative of the function with respect to 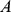 is derived as
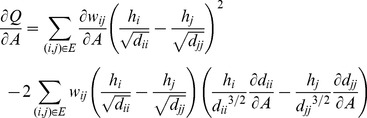
(13)


The derivatives of 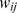 and 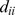 are computed as
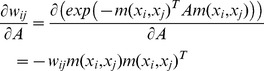
(14)




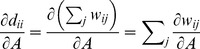
(15)With the derivative and initial metric 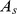 at the s-th iteration, the updated metric is obtained by
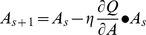
(16)where 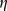 denotes the step length and 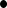 represents the Hadamard product. In order to preserve the positive semi-definite property of 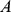, the updated 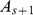 is further refined using the eigenvalue decomposition. The updated metric is decomposed as 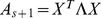, where 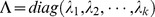 is a diagonal matrix of 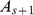
’s eigenvalues and the columns of 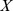 represents corresponding eigenvectors. A refined matrix is achieved by taking 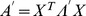, where 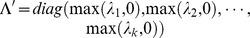.

The optimization will be solved with the alterations between the class label update stage and the metric update stage. The step size will be adapted to accelerate the convergence of the iteration process. If 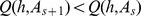, the step size will be doubled, and otherwise the step size will be reduced with keeping 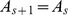 not changed. The convergence of our algorithm can be proved. At the stage of the class label update, the convex optimization always decreases the function value. As for the metric update stage, the gradient descent approach optimizes the metric with a reduced function value. With regard to the positive semi-definite of the normalized Laplacian matrix 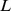, the cost function value has a lower bound of 0. The iterative process is thus guaranteed to converge. The iteration stops when the difference between cost function at the s-th and s+1-th iterations is smaller than a given threshold 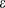.

### Assessment of Segmentation Accuracy

The accuracies of segmentation results were evaluated by the overlap accuracies. We utilized the popular dice similarity coefficient (DSC) for the assessment [47]. The evaluation metric is defined as
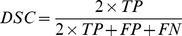
(17)where TP, FP and FN are the numbers of true positive, false positive and false negative voxels.

## Results

We validated the effectiveness and robustness of the proposed approach by experiments on both synthetic datasets and real brain DTI datasets from nine healthy subjects. In the experiments, we set the initial kernel matrix 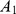 to a square diagonal matrix with entries of 1, which give equal weights of the element in the original distance vector. The step size 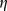 at the optimization was set to 0.01 and the threshold 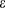 for stop criteria is set to 0.1. Our method usually stopped in less than 15 iterations. Our approach was compared with three classical metrics, including Euclidean, J-divergence and geodesic distances, in the graph based semi-supervised learning framework. The parameter 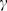 was set to 10 in these experiments using these predefined metrics. All the experiments were run on an Intel Core2 Duo desktop with 8GB RAM and 2×2.6 GHz CPU cores.

### Results on Synthetic Datasets

The proposed distance learning approach for DTI segmentation was first tested on the noise free synthetic dataset. In figure 1(a), the three color bars stand for the labels of the user inputs. The red color bar represents the label of the interested regions, and the other two green bars are the labels of the background. The proposed method was compared to three classical metrics, including Euclidean, J-divergence and geodesic metrics.

The three classical metrics obtained the same and incorrect segmentation results, as shown in figure 1(b), which only part of interested regions was extracted. In contrast, segmentation using our approach yielded the correct segmentation result, as shown in figure 1(c). The DSC value of our method was 1.0 and the values of other three approaches were 0.80.

**Figure 3:**
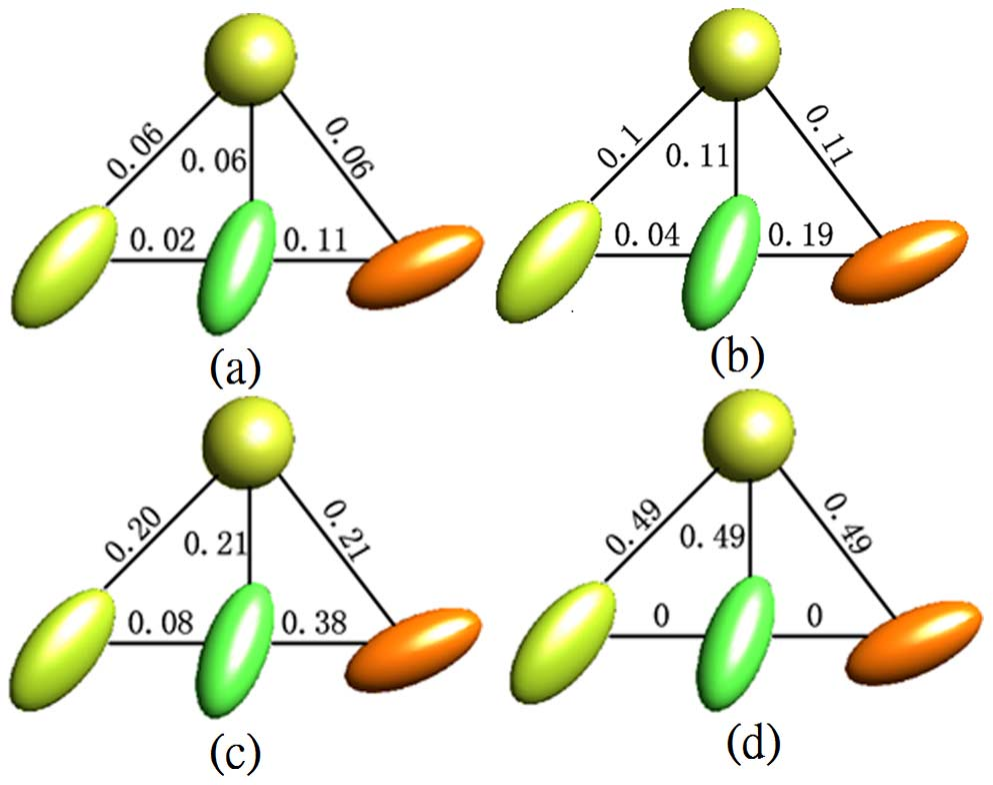
Different types of distances between diffusion tensors: (a) Euclidean metric, (b) J-divergence metric, (c) geodesic metric, (d) our learned metric.

The classical metrics give fixed contributions of the geometry and orientation features. However, the interested region had the same geometry feature but different principal orientations. This led to large Euclidean, J-divergence and geodesic distances between tensors in the region of interest, as shown in figure 3(a), (b) and (c) respectively. Due to the inappropriate metrics, the interested region could not be distinguished from the background. The reason for same segmentation results by these three metrics is the simple example, which has low variability of diffusion tensors of the synthetic dataset. The proposed method learned the relevant and irrelevant distances to construct a desired metric from supervisory information. With our learned metric, the tensors in the same label were close to each other, whereas the tensors in different label had large distances, as shown in figure 3(d). As a result, the interested region could be correctly extracted from the background while other three predefined metrics could not.

**Figure 4:**
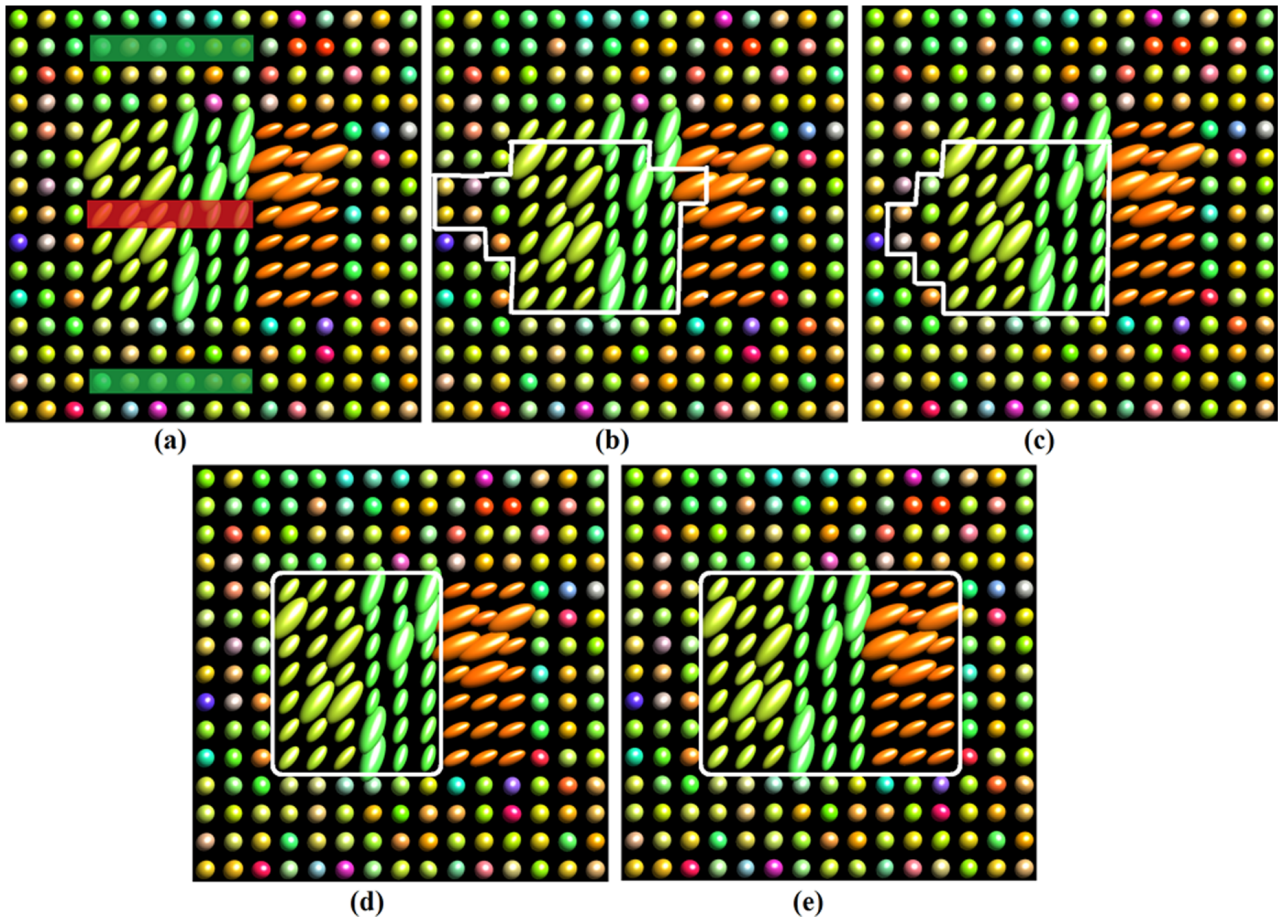
Segmentation results on the synthetic dataset with noise: (a) the noisy dataset, (b) Euclidean metric, (c) J-divergence metric, (d) geodesic metric, (e) our learned metric.

Further evaluation was performed on noisy synthetic diffusion tensor fields to assess the robustness. Figure 4(a) shows the noisy tensor fields with SNR of 15 and the labels. Figure 4(b), (c), (d) and (e) shows the segmentation results of the Euclidean, J-divergence, geodesic metrics and our learned metric respectively. The predefined metrics failed to recognize the regions of interest again. It can be seen that there are differences between the results of the three predefined metrics. This may be due to the higher variability of noisy tensor fields. Our learned distance obtained correct and accurate segmentations of the interested regions from the noisy diffusion tensor fields.

**Figure 5:**
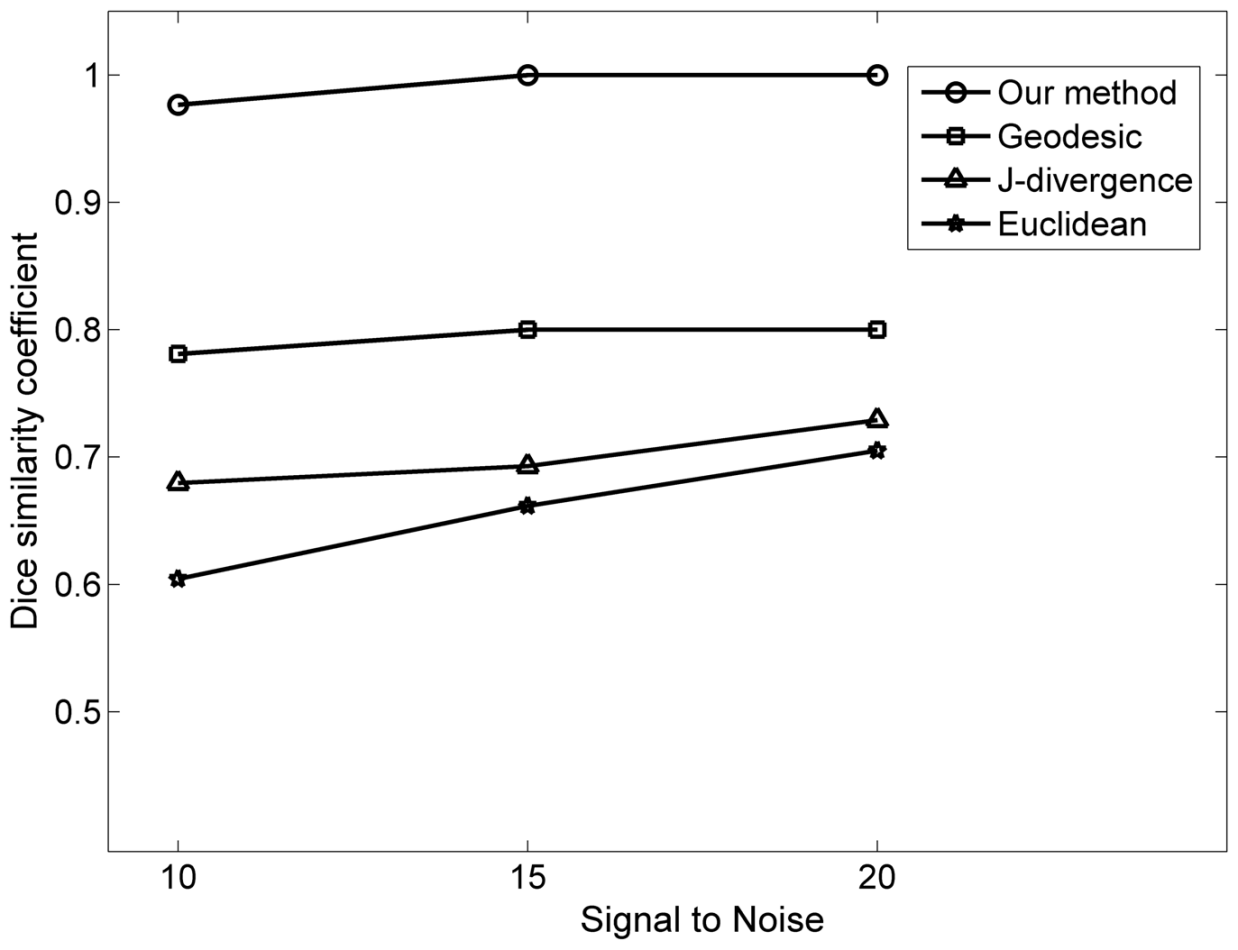
Dice similarity coefficients (DSC) of segmentation results using our approach and three predefined metrics on noisy synthetic datasets at different noise levels.

Figure 5 illustrates the DSC values of three predefined metrics and our approach on the noisy diffusion tensor fields at the SNR of 10, 15 and 20. Our approach achieved DSC value of 1 on the noisy datasets at SNR 15 and 20. There was slight decrease of DSC value on the datasets at SNR of 10. The DSC values of predefined metrics ranged from 0.6 to 0.8. The segmentation results of our approach had higher DSC values than that from the predefined metrics at the three noise levels. Segmentation results from the geodesic metric had relatively high DSC values than J-divergence and Euclidean metrics. These results demonstrate the robustness of the proposed distance metric learning approach.

### Results on Human Brain DTI Datasets

**Figure 6:**
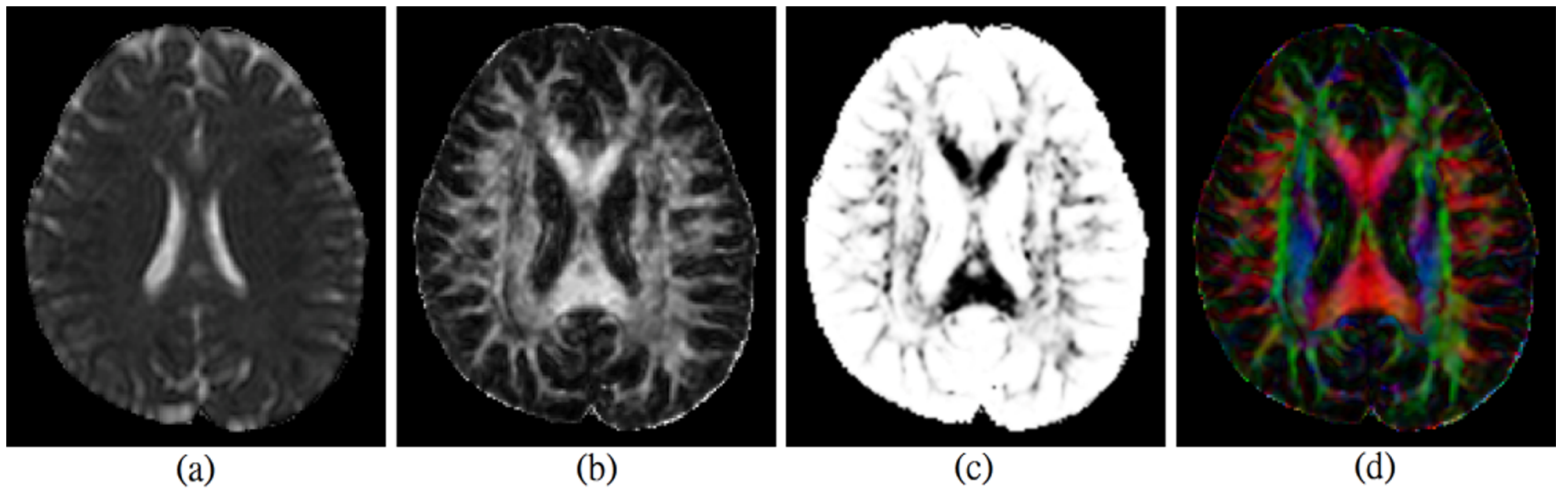
Scalar maps and orientation color maps of one axial slice from real dataset of one subject, (a) mean diffusion (MD) map, (b) fractional anisotropy (FA) map, (c) volume ratio (VR) map, (d) a color map for representing principal orientations of diffusion tensors.

We further evaluated our proposed approach on the human brain DTI datasets. Functional Magnetic Resonance Imaging of the Brain’s Diffusion toolbox [48] was utilized to perform the preprocessing of the DTI datasets. Distortion correction was first performed to remove the eddy currents and motion artifacts. Diffusion tensors were estimated using a least square method. Geometry and orientation parameters were then calculated for valid tensors at each voxel. Figure 6 shows three scalar maps and the color map for principal orientations of one axial slice. The scalar maps characterize different types of diffusion properties at each voxel. The color map represents the principal orientations of diffusion tensors at each voxel. Red, green and blue color refers to the orientations of left-right, anterior-posterior and superior-inferior.

To further evaluate the performance, we have performed the experiments of the corpus callosum segmentation in the brain DTI datasets. This important structure is the major fiber tract which connects the homologous cortical areas of the left and right hemisphere [49]. The three dimensional (3D) surface of the corpus callosum can be delineated with the powerful DTI, while it is difficult for the traditional MRI modalities. To reduce the computational time, we only kept voxels in the skull in the experiments. Since there was no ground truth of corpus callosum, manual segmentations were provided as the ground truth for comparisons. Manual segmentations were performed by a research associate who had 3 years of experience in MR measurement. The segmentation results were validated by an expert radiologist, who had over 10 years of experience in dealing with brain anatomy.

**Figure 7:**
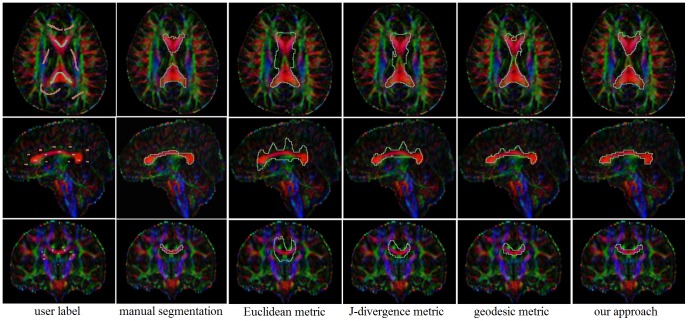
Segmentation results of the corpus callosum from brain DTI of one subject. Axial (1st row), sagittal (2nd row) and coronal (3rd row) views are showed. The 1st column is the user labels. The 2rd column is the manual segmentation results. The 3rd, 4th and 5th columns are results corresponding to Euclidean, J-divergence and geodesic metrics. The 6th column shows results of our proposed method.

**Figure 8:**
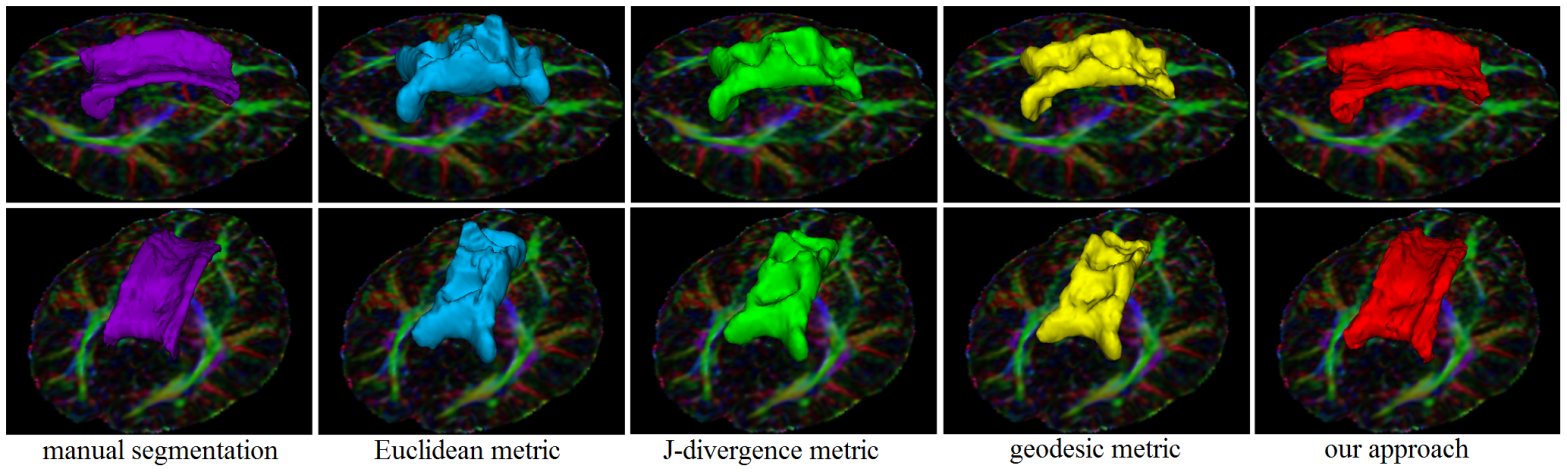
Surfaces of the corpus callosum segmentation: manual segmentation (violet), Euclidean metric (blue), J- divergence metric (green) and geodesic metric (yellow) and our learned metric (red).

**Figure 9:**
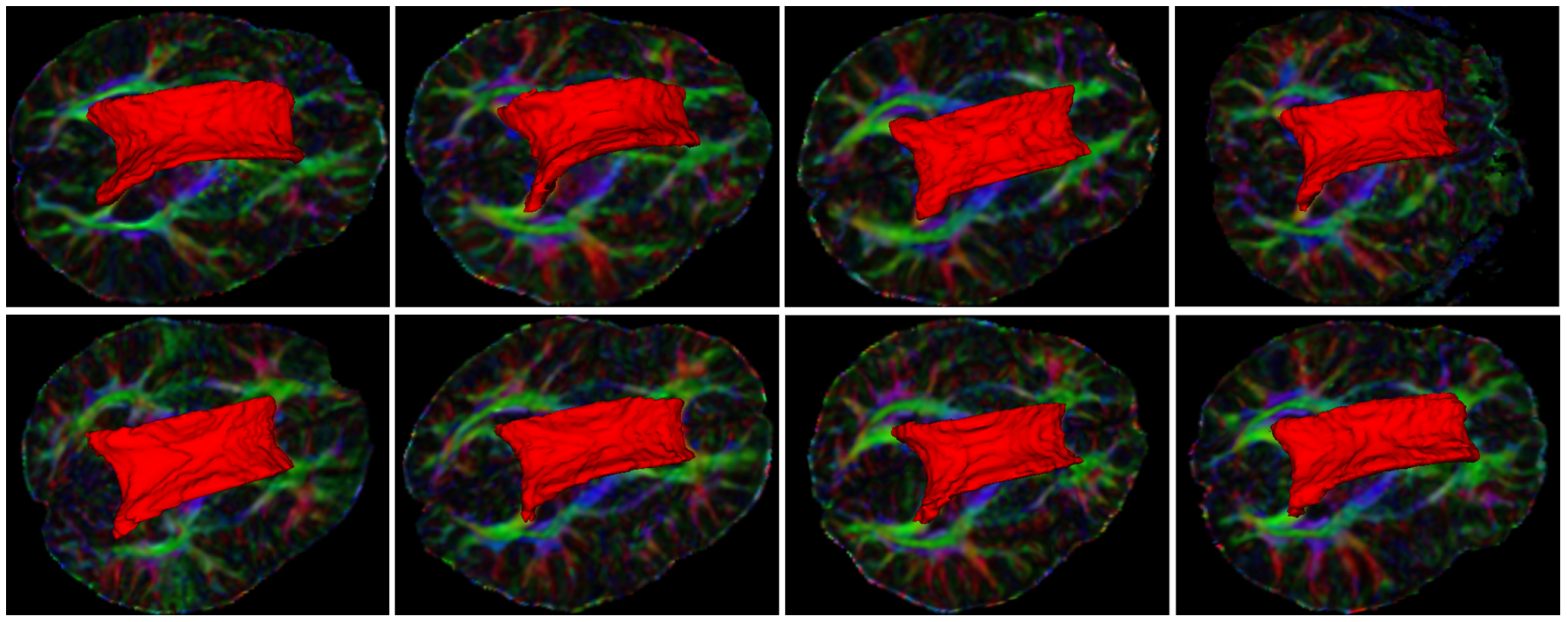
Surfaces of the corpus callosum segmentation results from another 8 datasets using our proposed approach.

Figure 7 shows the experiment results of the corpus callosum segmentation on one brain DTI dataset. The first column shows the provided labels on the colored map at multiple views. Initialized labels of the corpus callosum and the background were manually defined at a few axial slices with the FA map. The second column shows the manual segmentation results. The third, fourth and fifth columns show the boundaries of the segmentation results on axial, sagittal and coronal views by the Euclidean, J-divergence and geodesic metric respectively. The last column illustrates the boundary of the corpus callosum segmentation results using our approach. With the help of 3D Slicer tool [50], we visualized the 3D surfaces of the manual segmentation (violet), segmentation results of Euclidean (blue), J-divergence (green), geodesic metric (yellow) and our approach (red), as shown in figure 8. As manual segmentation for reference, our approach yielded better segmentation results compared with the classical metrics. The boundary of the corpus callosum is correctly delineated with the learned metric. The classical metrics are not enough discriminative to take over the boundary at some locations. Figure 9 shows the 3D surfaces of the segmentation results for DTI datasets from another eight subjects. Labels of the interested regions and the background were defined similar to the above experiment.

**Figure 10:**
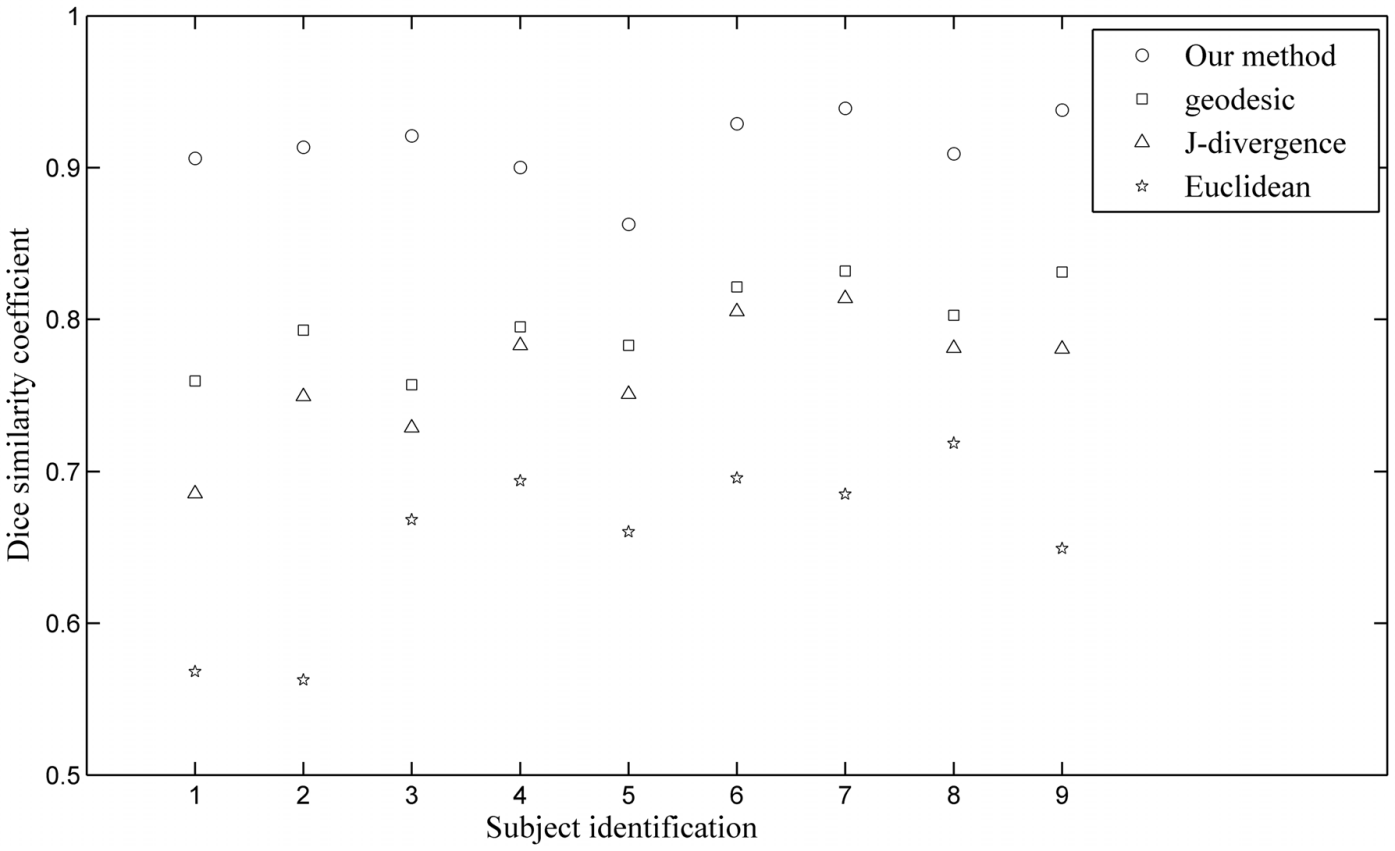
Dice similarity coefficients (DSC) of corpus callosum segmentations using our approach and three predefined metrics on all 9 subjects.

Figure 10 shows the DSC values of corpus callosum segmentation using our approach and predefined metrics for each subject. Our method achieved relatively high accuracies with DSC values around 0.90. The DSC values of the J-divergence and geodesic metrics were ranged from about 0.70 to 0.80, and the geodesic metric had relatively higher DSC values. The Euclidean approaches obtained the lowest DSC values for each subject.

Both qualitative and quantitative experiments demonstrated the superiority of our proposed approach compared to three classical metrics in the graph based semi-supervised learning framework. The straightforward Euclidean distance doesn’t consider the physical meaning of the diffusion tensor. The J-divergence metric and geodesic metric characterize the distance between tensors using the difference of their corresponding Gaussian distributions. Therefore, they obtained better results than Euclidean distance. However, the classical metrics provide fixed weights of geometry and orientation distances, which could not well differentiate the corpus callosum from the background. In contrast, our approach could learn an optimal mapping over the selected geometry and orientation distances based on the supervisory information. Under the learned metric, the corpus callosum can be correctly extracted from the complex surrounding structures.

The computational time of segmentation corpus callosum for one dataset was 5, 9 and 10 minutes for Euclidean, J-divergence, geodesic metrics respectively. Our approach required about one hour to learn an optimal metric for the segmentation. The computational time of metric learning is longer than the predefined metrics.

## Discussion

The powerful DTI technique can provide wealthy diffusion information, which can help distinguishing different anatomical structures and white matter tracts. Plenty of studies have developed many predefined metrics for different segmentation tasks in the last decade [12], [13], [15], [19], [42], [43], [51]–[54]. In this study, we presented to learn an optimal metric adaptive to the DTI segmentation application. To the best of our knowledge, this is the first time to learn metrics between diffusion tensors for DTI segmentation.

Learning metrics has several advantages over predefined metrics. An optimal distance metric can be automatically learned, which guarantee the closet match to the true target of interest in particular applications. In contrast, predefined metrics require adequate professional knowledge to obtain the properties of interested anatomical structures for different tasks. Tuning parameters are always performed on a large number of datasets Moreover, the parameters obtained from one group of datasets may not be optimal for another group due to difference in subject anatomies [55], image parameters [56], and even the scanner types [57]. The characteristics of brain structures have been found to be altered with normal aging [55], [58]. The diffusion tensor imaging can also be influenced by imaging parameters and MR scanners [56], [57]. By contrast, the distance metric learning is not susceptible to those factors with our adopted approach.

In this paper, we selected several geometry and orientation features to formulate the original discriminative distance. In our study, a nonlinear mapping over the original distance was learned to construct an optimal metric. Feature selection highly contributes the improvement of the segmentation performance. The three geometry features, including MD, FA and VR, have also been frequently used for differentiating different anatomical structures [16], [17]. The orientation feature captures the principal orientation of the water diffusion, which has been demonstrated to be able to distinguish several anatomical structures and white matter tracts [6], [18], [37]. However, the orientation distance may have limitations for some special applications. It may be not able to distinguish tensors with homogeneity of orientation, and justify the branching and crossing of white matter fibers. In future work, we will explore to develop other types of distances to overcome these limitations.

The distance metric learning was performed in a graph based semi-supervised learning approach. A large number of approaches [20], [21], [23], [25], [27], [28], [38], [45] have been developed to learn metric in machine learning and pattern recognition tasks. In future study, other distance metric learning approaches will be investigated with different type of segmentation methods, such as level set approach and atlas based method [59]. In addition, the distance metric learning will be explored to be applied in other DTI processing and analysis, such as image visualization, registration, interpolation, understanding and template construction.

One possible limitation of our approach is the relatively high computational time compared to predefined metrics. In the experiments, relatively longer time was required for our approach than segmentations by the predefined metrics. More time is required to optimize the metrics to an optimal one for an accurate segmentation. The operations in the optimization process have high potential to be accelerated using the advanced graphic processing unit. Besides, the learned metrics can be propagated to generate a good initial metric for a new dataset due to the similar properties of segmenting the same structure. A good initial metric could accelerate the convergence with reduced number of iterations, which thus decreases the computational time.

## Conclusion

In this paper, we have developed an effective and robust approach to learn adaptive distance metrics for DTI segmentation by a graph based semi-supervised learning model. An original discriminative distance vector was formulated with combination of geometry and orientation distances. A nonlinear mapping over the original distance was then optimized to construct an optimal metric with the graph based semi-supervised learning model. The constructed optimization problem was efficiently solved with a gradient descend approach. The performance of the proposed approach was evaluated on both synthetic and real DTI datasets. Experiments on nine human brain datasets were performed to demonstrate the robustness and reproducibility. The superiority of our approach was validated by comparing three classical metrics in the graph based semi-supervised learning framework.
